# Abnormal inhibition of osteoclastogenesis by mesenchymal stem cells through the miR-4284/CXCL5 axis in ankylosing spondylitis

**DOI:** 10.1038/s41419-019-1448-x

**Published:** 2019-02-25

**Authors:** Wenjie Liu, Peng Wang, Zhongyu Xie, Shan Wang, Mengjun Ma, Jinteng Li, Ming Li, Shuizhong Cen, Su’an Tang, Guan Zheng, Guiwen Ye, Xiaohua Wu, Yanfeng Wu, Huiyong Shen

**Affiliations:** 1 Department of Orthopedics, Sun Yat-sen Memorial Hospital, Sun Yat-sen University, Guangzhou, 510120 People’s Republic of China; 2 Center for Biotherapy, Sun Yat-sen Memorial Hospital, Sun Yat-sen University, Guangzhou, 510120 People’s Republic of China

## Abstract

Ankylosing spondylitis (AS) is a common inflammatory autoimmune disease, characterized by pathological osteogenesis. Mesenchymal stem cells (MSCs), as the main source of osteoblasts, participate in bone remodeling not only through differentiation into osteoblasts but also through indirect regulation of osteoclastogenesis. Our previous study indicated that the stronger osteogenic differentiation of MSCs from AS patients (ASMSCs) involved in pathological osteogenesis. However, whether there is any abnormality in the regulation of osteoclastogenesis by ASMSCs remains unclear. In this study, ASMSCs or MSCs from healthy donors (HDMSCs) were co-cultured with CD14 + monocytes in osteoclast induction medium. Our results demonstrated that ASMSCs exhibited a stronger capacity to inhibit osteoclastogenesis than HDMSCs. To explore underlying mechanisms, cytokine array assays were performed, showing that ASMSCs secreted more CXCL5 than HDMSCs, which was confirmed by enzyme-linked immunosorbent assays. Moreover, inhibition of osteoclastogenesis by ASMSCs was recovered by decreasing CXCL5. Besides, the inhibitory effect of CXCL5 on osteoclastogenesis was confirmed by exogenous addition. Bioinformatics analysis was applied to find the interaction between miR-4284 and CXCL5, which was verified by luciferase reporter assays. Furthermore, we used miR-4284 inhibitors or mimics to prove that the expression of CXCL5 was regulated by miR-4284. Further analysis showed that downregulation of miR-4284 in MSCs resulted in increase of CXCL5, markedly inhibiting osteoclastogenesis, whereas upregulation of miR-4284 in MSCs had the opposite effect. Our findings indicate that ASMSCs exhibit a stronger capacity to inhibit osteoclastogenesis than HDMSCs through the miR-4284/CXCL5 axis, which provide a new perspective on the mechanism of pathologic osteogenesis in AS.

## Introduction

Ankylosing spondylitis (AS) is a common inflammatory autoimmune disease that mainly affects the axial skeleton^1^. Although the pathogenesis of AS remains unknown, genetic, environmental, and immunological factors may be involved^2,3^. Pathological osteogenesis is one of the central features of AS^4^, though the mechanism is still unclear. Early diagnosis of pathological osteogenesis is currently difficult, and successful therapy for pathological osteogenesis has not yet been defined^5^. As many patients ultimately develop spinal ankylosis or hip joint ankylosis, which seriously affects quality of life^4^, it is important to study the pathogenesis of the osteogenesis that occurs in AS and to explore effective methods for early diagnosis and treatment.

Mesenchymal stem cells (MSCs) are the main source of osteoblasts^6^. We previously found that MSCs from AS patients (ASMSCs) displayed a stronger capacity to differentiate into osteoblasts than MSCs from healthy donors (HDMSCs), indicating the involvement of ASMSCs in pathological osteogenesis^7^. Osteoblasts and osteoclasts are the two key types of cells in bone remodeling^8–10^; MSCs participate in bone remodeling not only through differentiation into osteoblasts but also through indirect regulation of osteoclastogenesis^11,12^. However, whether there is any abnormality in the regulation of osteoclastogenesis by ASMSCs remains unclear.

Human osteoclasts are largely derived from CD14^+^ monocytes^13^, and stimulation with monocyte colony-stimulating factor (M-CSF) and receptor activator of NF-κB ligand (RANKL) can cause monocytes to differentiate into osteoclasts^14^. Nuclear factor of activated T cells c1 (NFATc1) is the master transcription factor for osteoclastogenesis^14^. The major function of osteoclasts is bone resorption, which primarily depends on tartrate-resistant acid phosphatase (TRAP) and cathepsin K (CTSK)^15^. Many inflammatory cytokines regulate osteoclastogenesis^16^. For example, IL-4 and IL-10 inhibit osteoclastogenesis, whereas SDF-1 and MCP-1 stimulate osteoclastogenesis^16–18^. Previous studies have shown that CXCL5 has a strong effect on neutrophil recruitment and is involved in a variety of inflammatory diseases, such as rheumatoid arthritis (RA) and pediatric ulcerative colitis^19–21^, yet its role in AS remains unknown.

MicroRNAs (miRNAs) are small noncoding RNAs that regulate gene expression by causing mRNA degradation or translational inhibition^22^ through interaction via a seed sequence (2–7 nucleotide) in the 3′-untranslated region of the target mRNA^22^. miRNAs play an important role in many processes, including cell proliferation, differentiation, and apoptosis^16^. Abnormal expression of miRNAs is involved in the development of many autoimmune diseases, such as RA and systemic lupus erythematosus^23^. For example, compared to healthy donors (HDs), higher levels of miR-146a, miR-125a, miR-151a, and miR-22 and lower levels of miR-150 and miR-451a have been found in AS patients, and these miRNAs can serve as biomarkers of disease activity in AS^24^. These results suggest that miRNAs may participate in the pathology of AS.

In this study, we investigated the effect of ASMSCs compared with HDMSCs on osteoclastogenesis and explored the mechanism of abnormal inhibition of osteoclastogenesis by MSCs in AS. Our results demonstrate that the ability to inhibit osteoclastogenesis is enhanced in ASMSCs compared with HDMSCs through secretion of CXCL5, which can be regulated by miR-4284. These findings provide a new perspective on the mechanism of pathologic osteogenesis in AS.

## Materials and methods

### Cell isolation and culture

In this study, 30 HDs and 30 AS patients who satisfied the modified New York criteria^25^ were recruited. Details of the study subjects were showed in Supplementary Table S1. The protocol was approved by the Ethics Committee of Sun Yat-sen Memorial Hospital (Sun Yat-sen University, Guangzhou, China), and informed consent was obtained from all subjects. MSCs were isolated and purified as previously described^7^ and cultured in Dulbecco’s modified Eagle’s medium (Gibco) containing 10% fetal bovine serum (Sijiqing Biological Engineering Material Company). The culture medium was replaced every 3 days.

Whole blood was obtained from HDs with the permission of the Ethics Committee of Sun Yat-sen Memorial Hospital. Peripheral blood mononuclear cells (PBMCs) were isolated from whole blood using density-gradient centrifugation as previously described^26^. CD14^+^ monocytes were isolated via positive selection of PBMCs labeled with magnetic-bead-conjugated anti-human CD14 mAbs according to the protocol provided by the manufacturer (Miltenyi Biotech). For assessment of CD14^+^ monocyte purity, cells were incubated with fluorescein isothiocyanate (FITC)-conjugated anti-CD14 mAb (BD Biosciences) on ice for 30 min, washed three times with phosphate-buffered saline (PBS) and detected using a BD Influx cell sorter (BD Biosciences). All cells were cultured at 37 °C in 5% CO_2_.

### Co-culture system for osteoclast differentiation

CD14^+^ monocytes (2.5 × 10^5^/cm^2^) were seeded in the lower wells and MSCs (monocytes: MSCs = 10:1) in the upper wells of transwell plates (0.4 µm pore size; Corning). For induction of osteoclast differentiation, CD14^+^ monocytes (2.5 × 10^5^/cm^2^) were cultured in α-minimum essential medium containing 10% fetal bovine serum supplemented with 25 ng/mL recombinant human M-CSF and 50 ng/mL recombinant human RANKL (both from Peprotech). The medium was replaced every 3 days.

### TRAP staining

Cells were washed three times with PBS on day 9 and stained for TRAP activity using the leukocyte acid phosphatase kit (Sigma) according to the manufacturer’s instructions. TRAP-positive cells with at least three nuclei were considered osteoclasts. At least nine fields of view covering the entire plate were assessed, and the mean number of osteoclasts was calculated.

### F-actin assay

Cells were fixed for 5 min with 4% paraformaldehyde on day 9 and washed extensively with PBS. The cells were then stained with FITC-conjugated phalloidin (Sigma) and 4’, 6-diamidino-2-phenylindole for 40 min at room temperature. Afterwards, the cells were washed three times with PBS and observed with an Axio Observer fluorescence microscope (Carl Zeiss).

### Bone resorption assay

For determination of the capacity of osteoclasts to resorb bone, CD14^+^ monocytes (2.5 × 10^5^/cm^2^) were plated onto bovine cortical slices in 24-well plates and cultured with MSCs in osteoclast differentiation medium for up to 15 days. To evaluate bone resorption, the slices were washed thoroughly with PBS and fixed with 2.5% glutaraldehyde for 15 min. The slices were then washed three times with PBS and treated with ultrasonication in 0.5 m NH_4_OH to remove adherent cells. After washing three times with PBS, the slices were gradient-dehydrated in 70, 95, and 100% ethanol, stained for 5 min with 1% (w/v) toluidine blue and washed five times with PBS. At least nine fields of view covering the entire slice were assessed, and the mean number of pit formations was calculated.

### Real-time quantitative reverse transcription–polymerase chain reaction (qRT-PCR)

Cells were washed three times with PBS, and total RNA was extracted after the addition of TRIzol (Invitrogen). Complementary DNA was transcribed from 1 µg of RNA using a PrimeScript RT reagent kit (TaKaRa) according to the instructions. qRT-PCR was carried out using a LightCycler®480 PCR system (Roche) with SYBR Premix Ex Taq (TaKaRa). The steps were described in our previously published research^27^. The procedure for the qRT-PCR assay was 95 °C for 30 s and 40 cycles at 95 °C for 5 s and 60 °C for 20 s. Each sample was performed in triplicate, and the mean mRNA level was calculated. To confirm specific amplification of targets, the melting curve for each sample was analyzed. GAPDH or U6 was used as internal control, and the relative expression of each gene was calculated using the 2^−ΔΔCt^ method. The forward and reverse primers used for target genes are available in Supplementary Table S2.

### Western blot analysis

Cells were lysed in RIPA buffer (Sigma) supplemented with 1% protease and phosphatase inhibitors (Roche) for 30 min on ice. The lysates were then centrifuged at 12,000 rpm for 30 min at 4 °C, and the proteins in the supernatant were quantified using a BCA assay kit (Sigma) according to the manufacturer’s instructions. Equal amounts of protein diluted in sodium dodecyl sulfate (SDS) loading buffer, separated by 10% SDS–polyacrylamide gel electrophoresis, and electrotransferred onto polyvinylidene fluoride membranes (EMD Millipore Billerica). The membranes were blocked with 5% skim milk in TBST at room temperature for 60 min and incubated overnight at 4 °C with primary antibodies against GAPDH, TRAP, CTSK, NFATc1, p65, phosphorylated p65, phosphorylated p38, p38, ERK-1/2, phosphorylated ERK-1/2, JNK, and phosphorylated JNK (each diluted 1:1000; Cell Signaling Technology). Afterwards, the membranes were washed three times with TBST and incubated with appropriate horseradish peroxidase (HRP)-conjugated secondary antibodies (diluted 1:3000; Santa Cruz Biotechnology) at room temperature for 60 min. The membranes were washed three times with TBST, and signals were detected using Immobilon Western Chemiluminescent HRP Substrate (Millipore).

### Cytokine array assay

MSCs (2.5 × 10^4^/cm^2^) were seeded into 12-plate wells. After 3 days, culture supernatants were collected from HDMSCs and ASMSCs, respectively. Then culture supernatants of MSCs were analyzed using Proteome Profiler Human XL Cytokine Array Kit (R&D Systems) according to the manufacturer’s instructions. For each assay, 500 µL of cell culture supernatant was used, and quantification of cytokine optical densities was obtained with HLImage + + software (Western Vision). Densitometry of signal intensities was performed to quantify differences in cytokines between ASMSCs and HDMSCs.

### Enzyme-linked immunosorbent assay (ELISA)

MSCs (2.5 × 10^4^/cm^2^) were seeded into 12-plate wells and supernatants of MSCs were harvested on day 3. The level of CXCL5 protein in the cell culture supernatant was quantitated using Human CXCL5 Quantikine ELISA Kit (R&D Systems) following the manufacturer’s protocol.

### Lentivirus construction and infection

A lentivirus encoding a short hairpin RNA (shRNA, GenePharma) for CXCL5 (sh-CXCL5) was constructed; the target sequence was 5′-AGAGCUGCGUUGCGUUUGUTT-3′. The sequence for the negative control was 5′-UUCUCCGAACGUGUCACGUTT-3′. For transfection, the lentivirus and polybrene (5 µg/mL) were added to the MSC culture medium for 24 h (MOI = 50).

### Exogenous CXCL5 assay

Recombinant human CXCL5 (R&D Systems) was added to the culture medium at different concentrations (1, 5, 25, and 125 ng/mL) every 3 days. Cells were grown in the presence of M-CSF (25 ng/mL) and RANKL (50 ng/mL). After 12 days, the cells were stained for TRAP and F-actin, and after 15 days, resorption pits on the slices were stained with toluidine blue.

### Luciferase reporter assay

A CXCL5 3′-UTR mutant with a mutated seed region for predicted miR-4284 binding sites was constructed and named CXCL5MUT. The synthesized wild-type (CXCL5WT) or mutant (CXCL5MUT) sequence was cloned into the pmirGLO vector containing the firefly luciferase structural gene. For transfection, 5000 MSCs per well were seeded onto 96-well plates, and each construct was co-transfected with miR-4284 mimics or a negative control using Lipofectamine 3000 Transfection Reagent (Invitrogen). After 36 h, cell lysates were harvested, and luciferase activity was determined using Dual-Luciferase® Reporter (DLR™) Assay System (Promega) according to the manufacturer’s instructions. The ratio of firefly to Renilla luciferase activity was calculated as the relative luciferase activity.

### Statistical analysis

Statistical analysis was performed using SPSS 20.0 software (Chicago, IL, USA). Comparisons between two groups were performed using Student’s *t* test. In addition, differences among three or more groups were analyzed by one-way analysis of variance. A value of *p* < 0.05 was considered significant.

## Results

### ASMSCs exhibit a stronger ability to inhibit osteoclastogenesis than HDMSCs

**Fig. 1:**
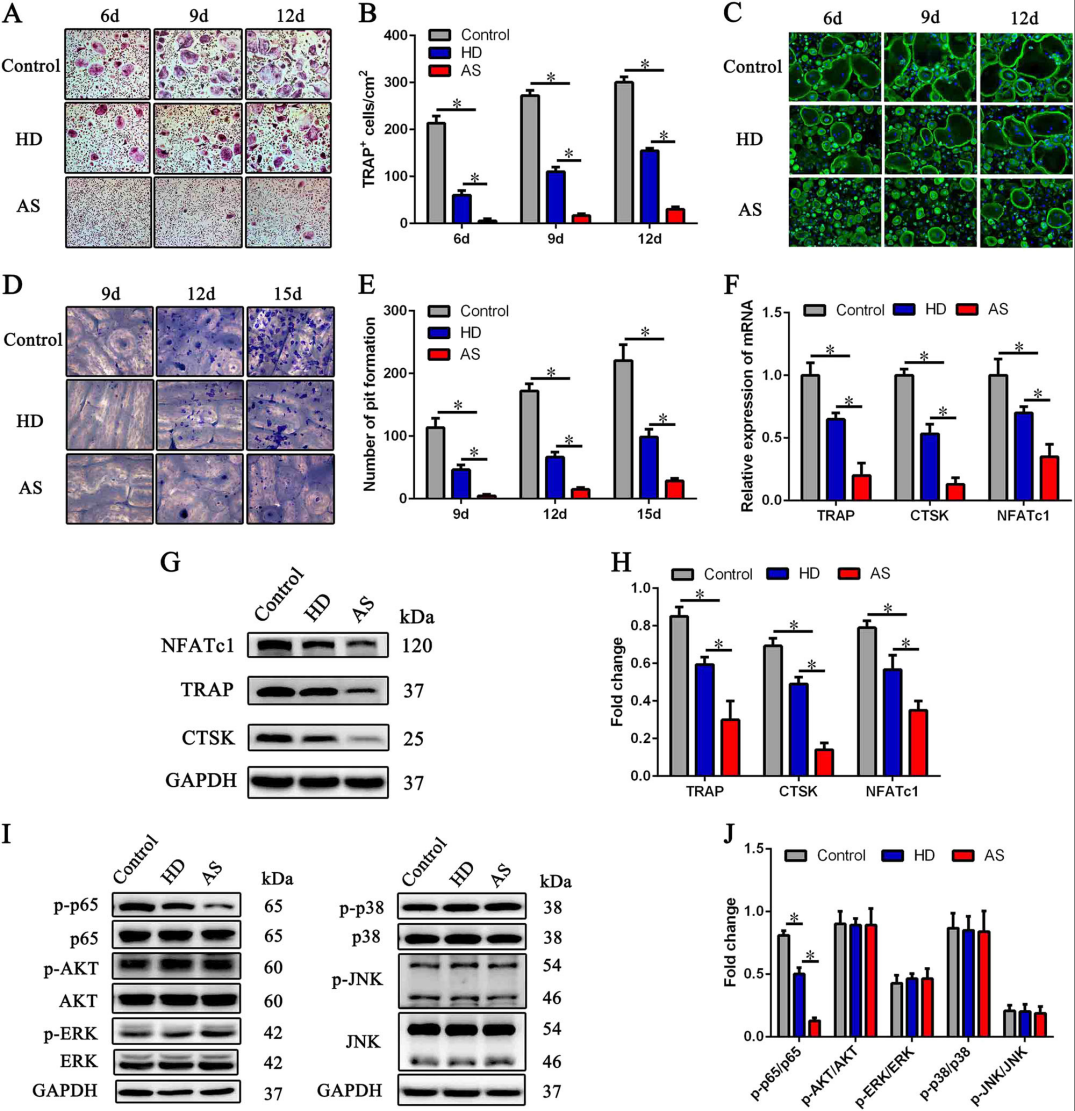
Inhibition of osteoclastogenesis by ASMSCs compared with HDMSCs. CD14 + monocytes were cultured with HDMSCs or ASMSCs in the presence of M-CSF and RANKL. **a** Representative images of TRAP staining of osteoclasts co-cultured at different time points (× 100). **b** The number of TRAP^+^ osteoclasts in each well from cultures at different time points is shown. **c** Representative images of osteoclasts stained with FITC-phalloidin at different time points (× 200). **d** Representative images for bone resorption assays at different time points ( × 200). Cells cultured with HDMSCs or ASMSCs on bovine cortical slides were stained with toluidine blue. **e** Pit formation on each slide was assessed. **f** mRNA expression levels of TRAP, CTSK, and NFATc1 in osteoclasts were determined by qPCR on day 9. **g** Protein levels of TRAP, CTSK, and NFATc1 in osteoclasts were determined by western blot analyses on day 9. **h** Quantitative data of TRAP, CTSK, and NFATc1 protein levels determined by western blot analyses are shown. **i** Activation of signaling pathways involved in osteoclastogenesis was determined by western blot analyses on day 9. **j** Quantitative data for activation of signaling pathways determined by western blot analyses are shown. Values are the mean ± SD of 30 samples per group. The results represent three independent experiments. *, *p* *<* 0.05; HDMSCs, mesenchymal stem cells from healthy donors; ASMSCs, mesenchymal stem cells from patients with ankylosing spondylitis

To investigate differences in the ability of HDMSCs and ASMSCs to inhibit osteoclastogenesis, we cultured CD14^+^ monocytes with MSCs; the medium was supplemented with M-CSF and RANKL to induce osteoclast differentiation. Purification of CD14^+^ monocytes was > 95%, as confirmed by flow cytometry (Supplementary Figure S1). TRAP staining assays were performed on days 6, 9, and 12, and the number of TRAP^+^ osteoclasts was significantly decreased after co-culture with MSCs, with fewer osteoclasts in the ASMSC group than that of HDMSC group (Fig. 1a). The TRAP staining assay quantification is shown in Fig. 1b. As the band of F-actin containing podosomes in osteoclasts participates in bone resorption, the F-actin assays indicated that these osteoclast-like cells can resorb bone (Fig. 1c). Furthermore, pit formation was decreased after culture with MSCs (Fig. 1d). Most notably, CD14^+^ monocytes cultured with ASMSCs exhibit difficulty in differentiating into osteoclasts and causing decreased bone resorption compared with cells cultured with HDMSCs (Fig. 1d). The bone resorption assay results are summarized in Fig. 1e. These findings indicate that ASMSCs have a stronger capacity than HDMSCs to inhibit osteoclastogenesis.

As presented in Fig. 1f, qRT-PCR revealed that osteoclasts cultured with ASMSCs expressed lower levels of TRAP, CTSK, and NFATc1 than cells cultured with HDMSCs, and the results of the western blot analyses were consistent (Fig. 1g, h). Moreover, osteoclasts cultured with ASMSCs expressed lower levels of phosphorylated p65 than osteoclasts cultured with HDMSCs, though no differences in JNK, p38, AKT, and ERK pathway components were found (Fig. 1i, j). These results suggest that the p65 pathway is essential for the ASMSC-mediated inhibition of osteoclastogenesis.

### ASMSCs secrete more CXCL5 than do HDMSCs

**Fig. 2:**
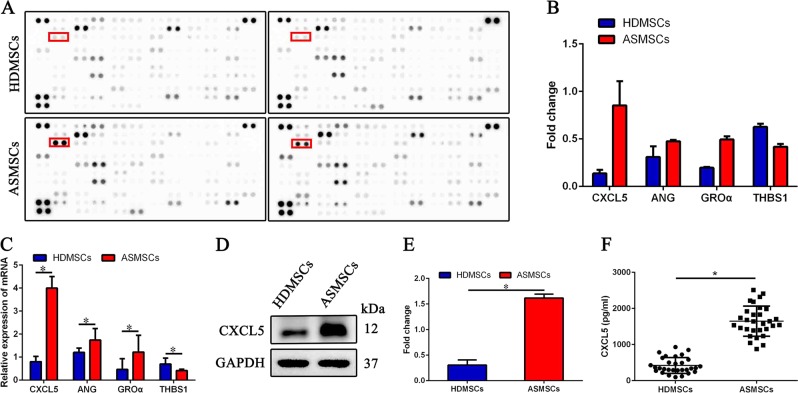
ASMSCs secrete more CXCL5 than do HDMSCs. **a**, **b** Culture supernatants collected from MSCs on day 3 were analyzed using a Human XL Cytokine Array Kit. Several cytokines with differential expression were found in the culture supernatants of ASMSCs, including CXCL5, ANG, GROα, and THBS1. **c** Expression levels of these cytokines in ASMSCs and HDMSCs were confirmed by qRT-PCR. **d** Western blot analysis was performed to detect the protein level of CXCL5 in ASMSCs compared with HDMSCs. **e** Quantitative data for western blot analyses are shown. **f** CXCL5 secreted from HDMSCs and ASMSCs was measured by ELISA on day 3. Data are presented as the mean ± SD (*n* = 30). The results represent three independent experiments. *, *p* *<* 0.05; HDMSCs, mesenchymal stem cells from healthy donors; ASMSCs, mesenchymal stem cells from patients with ankylosing spondylitis; ANG, angiogenin; THBS1, thrombospondin-1

To identify factors involved in the excessive inhibition of osteoclastogenesis by ASMSCs, we analyzed culture supernatants using a Human XL Cytokine Array Kit. Several cytokines with abnormal expression were found in the culture supernatants of ASMSCs, including CXCL5, angiogenin (ANG), GROα and thrombospondin-1 (THBS1) (Fig. 2a, b). qRT-PCR assays were performed to confirm expression levels of these cytokines in ASMSCs and HDMSCs. Of note, the most prominent increase was found for CXCL5 (Fig. 2c), and the results of western blot assays were consistent with these qRT-PCR results (Fig. 2d, e). Increased release of CXCL5 from ASMSCs was also confirmed by ELISA (Fig. 2f).

### Effect of sh-CXCL5 on the inhibition of osteoclastogenesis by MSCs

**Fig. 3:**
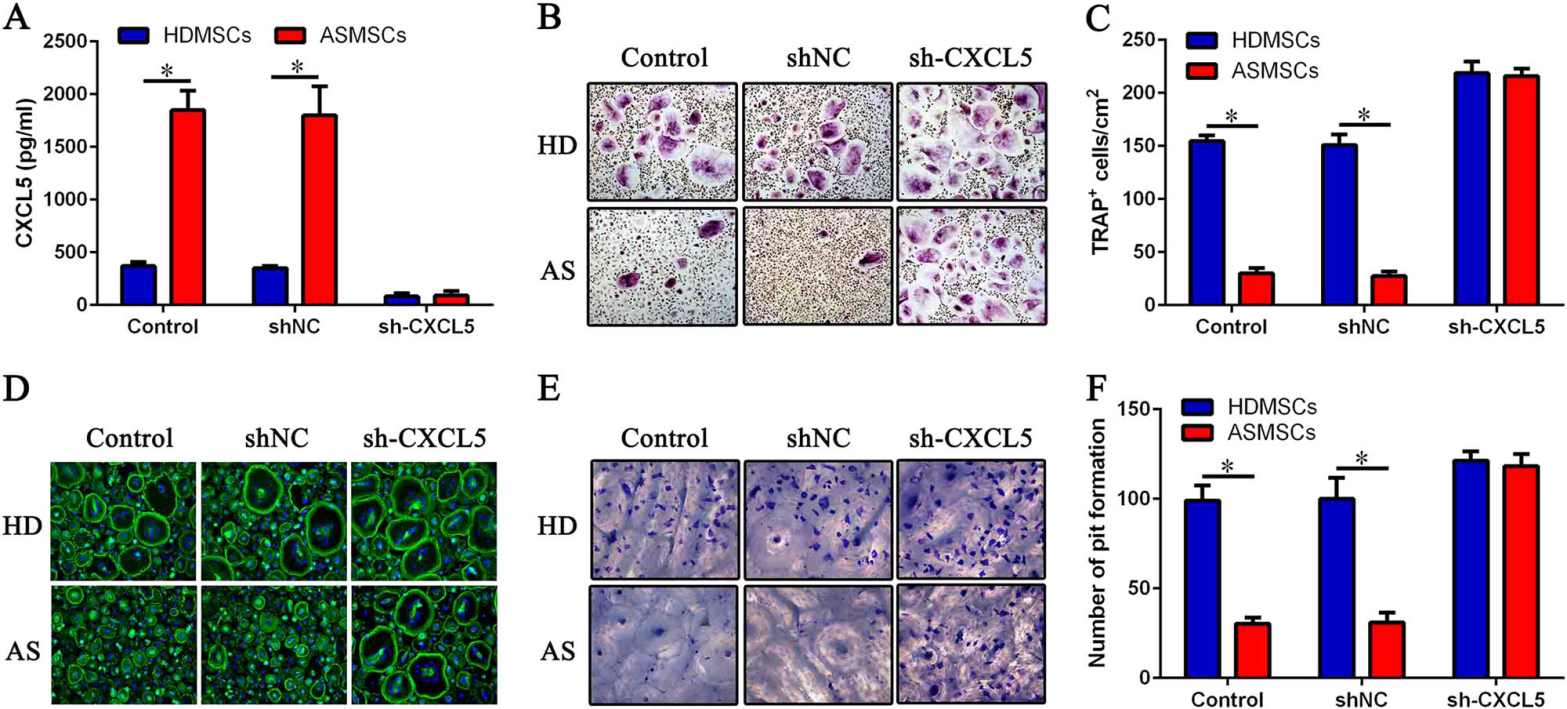
Effect of sh-CXCL5 on the inhibition of osteoclastogenesis by MSCs. CD14 + monocytes were cultured with HDMSCs or ASMSCs after knocking down CXCL5 in the presence of M-CSF and RANKL. **a** Secretion of CXCL5 from MSCs was detected by ELISA on day 3 after transfection with sh-CXCL5. **b** Representative images of TRAP staining (× 100). **c** The number of TRAP^+^ osteoclasts in each well is shown. **d** Representative images of F-actin assays (× 200). **e** Representative images of bone resorption assays (× 200). **f** Pit formation on each slide was assessed. Data are presented as the mean ± SD (*n* = 30). The results represent three independent experiments. *, *p* *<* 0.05; HDMSCs, mesenchymal stem cells from healthy donors; ASMSCs, mesenchymal stem cells from patients with ankylosing spondylitis

To determine whether CXCL5 is the key cytokine for the observed increase in osteoclastogenesis inhibition by ASMSCs, we constructed a lentivirus encoding a short hairpin RNA targeting CXCL5 (sh-CXCL5). Both HDMSCs and ASMSCs were effectively transfected with the lentivirus (Supplementary Figure S2), and production of CXCL5 was reduced by at least 80% (Fig. 3a). After CXCL5 knockdown, the number of osteoclasts increased, with no notable difference in the number of osteoclasts between HDMSCs and ASMSCs (Fig. 3b, c). The results of F-actin and bone resorption assays were consistent with the results of TRAP staining (Fig. 3d–f), indicating that elevated CXCL5 was the main cause of the increased inhibition of osteoclastogenesis by ASMSCs.

### Exogenous CXCL5 inhibits osteoclastogenesis in a dose-dependent manner through the p65 pathway

**Fig. 4:**
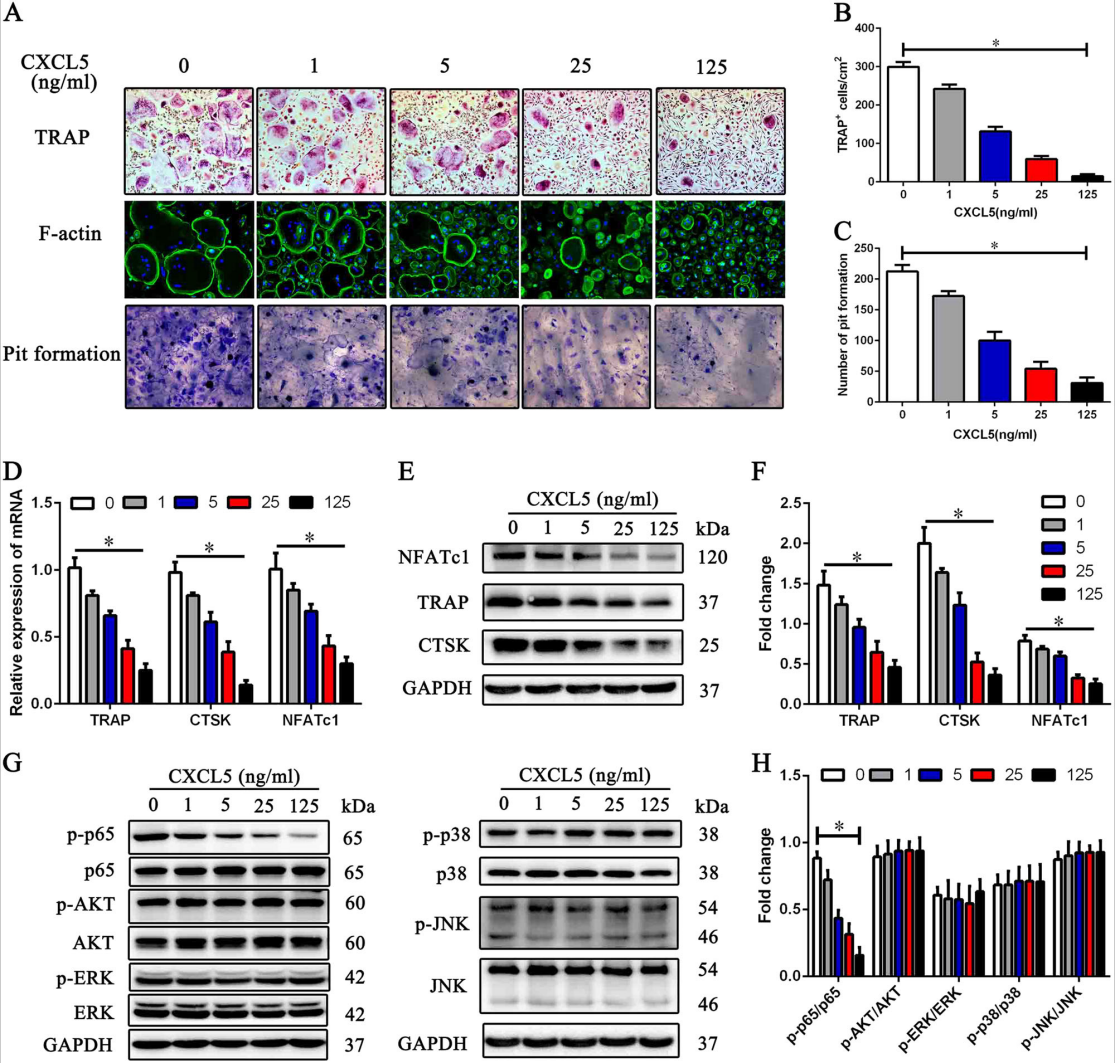
Effect of exogenous CXCL5 on osteoclastogenesis. **a** Representative images of TRAP staining (× 100), F-actin and bone resorption assays (× 200) of osteoclasts treated with the indicated doses of CXCL5 under osteoclastogenic conditions. **b** The number of TRAP^+^ osteoclasts in each well on day 9 is shown. **c** Pit formation on each slide on day 15 was assessed. **d** mRNA expression levels of TRAP, CTSK, and NFATc1 were determined by qRT-PCR on day 9. **e** Protein levels of TRAP, CTSK, and NFATc1 were determined by western blot analysis on day 9. **f** Quantitative data for TRAP, CTSK, and NFATc1 protein levels determined by western blot analyses are shown. **g** Activation of signaling pathways involved in osteoclastogenesis was determined by western blot analyses on day 9. **h** Quantitative data for activation of signaling pathways determined by western blot analyses are shown. Data are presented as the mean ± SD (*n* = 18). The results represent three independent experiments. *, *p* *<* 0.05

To investigate the effect of CXCL5 on osteoclastogenesis, we cultured CD14^+^ monocytes under osteoclastogenic conditions in the continuous presence of CXCL5 at different concentrations (0, 1, 5, 25, 125 ng/mL). As determined by TRAP staining, the number of TRAP^+^ osteoclasts gradually decreased as the concentration of CXCL5 increased (Fig. 4a, b). F-actin and bone resorption assays showed that bone resorption capacity also decreased with increasing concentration of CXCL5 (Fig. 4a, c). These data demonstrate that CXCL5 inhibits osteoclastogenesis in a dose-dependent manner.

qRT-PCR and western blot analyses showed that the expression levels of TRAP, CTSK, and NFATc1 in osteoclasts were decreased after stimulation with CXCL5 (Fig. 4d, f). In addition, phosphorylation of p65 was markedly decreased after stimulation with CXCL5, though the JNK, p38, AKT, and ERK pathways were not affected (Fig. 4g, h). These results show that CXCL5 inhibits osteoclastogenesis through the p65 pathway.

### miR-4284 is decreased in ASMSCs and regulates expression of CXCL5

**Fig. 5:**
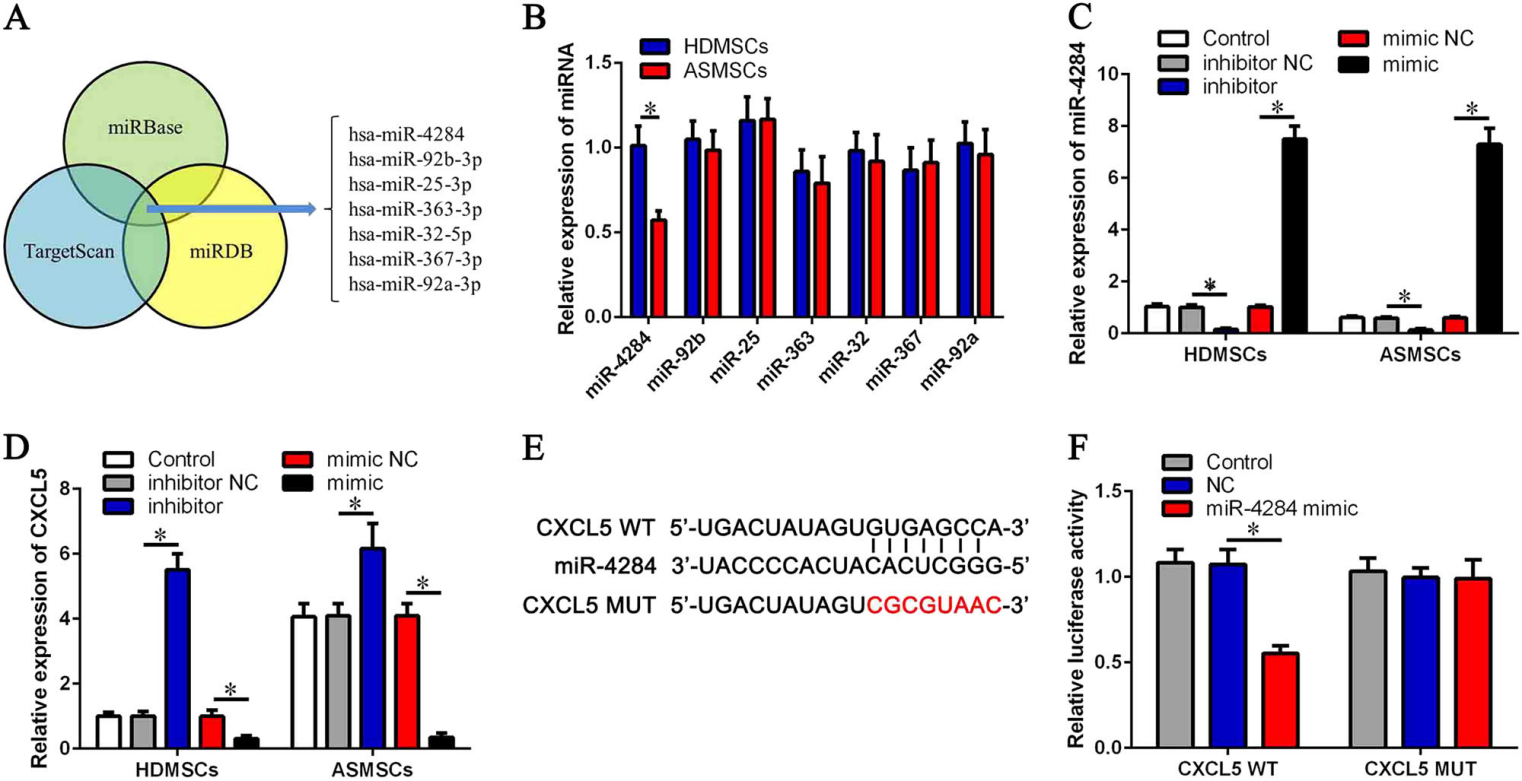
miR-4284 is decreased in ASMSCs and regulates expression of CXCL5. **a** Bioinformatics analysis in three databases (TargetScan, miRBase, and miRDB) to identify possible miRNAs targeting CXCL5. Seven miRNAs with the highest scores for targeting CXCL5 are shown. **b** Expression levels of miRNAs in HDMSCs and ASMSCs were determined by qRT-PCR. **c** Expression levels of miR-4284 in HDMSCs and ASMSCs after treatment with miR-4284 inhibitor or mimic. **d** Expression levels of CXCL5 in HDMSCs and ASMSCs after treatment with miR-4284 inhibitor or mimic. **e** Possible binding sites between miR-4284 and CXCL5 are shown, and a mutant (MUT) CXCL5 site was constructed. **f** Binding sites were confirmed by luciferase assays. The miR-4284 mimic decreased the luciferase activity of wild-type (WT) CXCL5 but had no impact on MUT CXCL5. Data are presented as the mean ± SD (*n* = 30). The results represent three independent experiments. *, *p* *<* 0.05; HDMSCs, mesenchymal stem cells from healthy donors; ASMSCs, mesenchymal stem cells from patients with ankylosing spondylitis

miRNAs can regulate the paracrine effects of MSCs and also participate in the pathogenesis of AS. To further explore the upstream molecular mechanism of upregulated CXCL5 expression in ASMSCs, we selected possible miRNAs targeting CXCL5 using bioinformatics analysis of three databases (TargetScan, miRBase, and miRDB). Seven miRNAs had the highest scores for CXCL5 targeting (Fig. 5a), and qRT-PCR showed only miR-4284 to be downregulated in ASMSCs (Fig. 5b). To investigate whether miR-4284 regulates expression of CXCL5, we transfected MSCs with a miR-4284 inhibitor or mimic and found that CXCL5 was upregulated in the miR-4284 inhibitor group and downregulated in the miR-4284 mimic group (Fig. 5c, d). Moreover, there was no difference in CXCL5 expression between HDMSCs and ASMSCs transfected with miR-4284 inhibitor or mimic (Fig. 5c, d). We next performed luciferase reporter assays to further verify the direct interaction between miR-4284 and CXCL5 and predicted binding sites in the 3′-UTR of CXCL5. Using WT and MUT CXCL5 (Fig. 5e) constructs, luciferase reporter assays revealed significantly decreased luciferase activity for WT CXCL5 owing to the miR-4284 mimic, with no impact on MUT CXCL5 (Fig. 5f). These results demonstrate that miR-4284 is downregulated in ASMSCs, inducing elevated CXCL5 expression, which may influence osteoclastogenesis.

### miR-4284 can regulate MSC-mediated inhibition of osteoclastogenesis

**Fig. 6:**
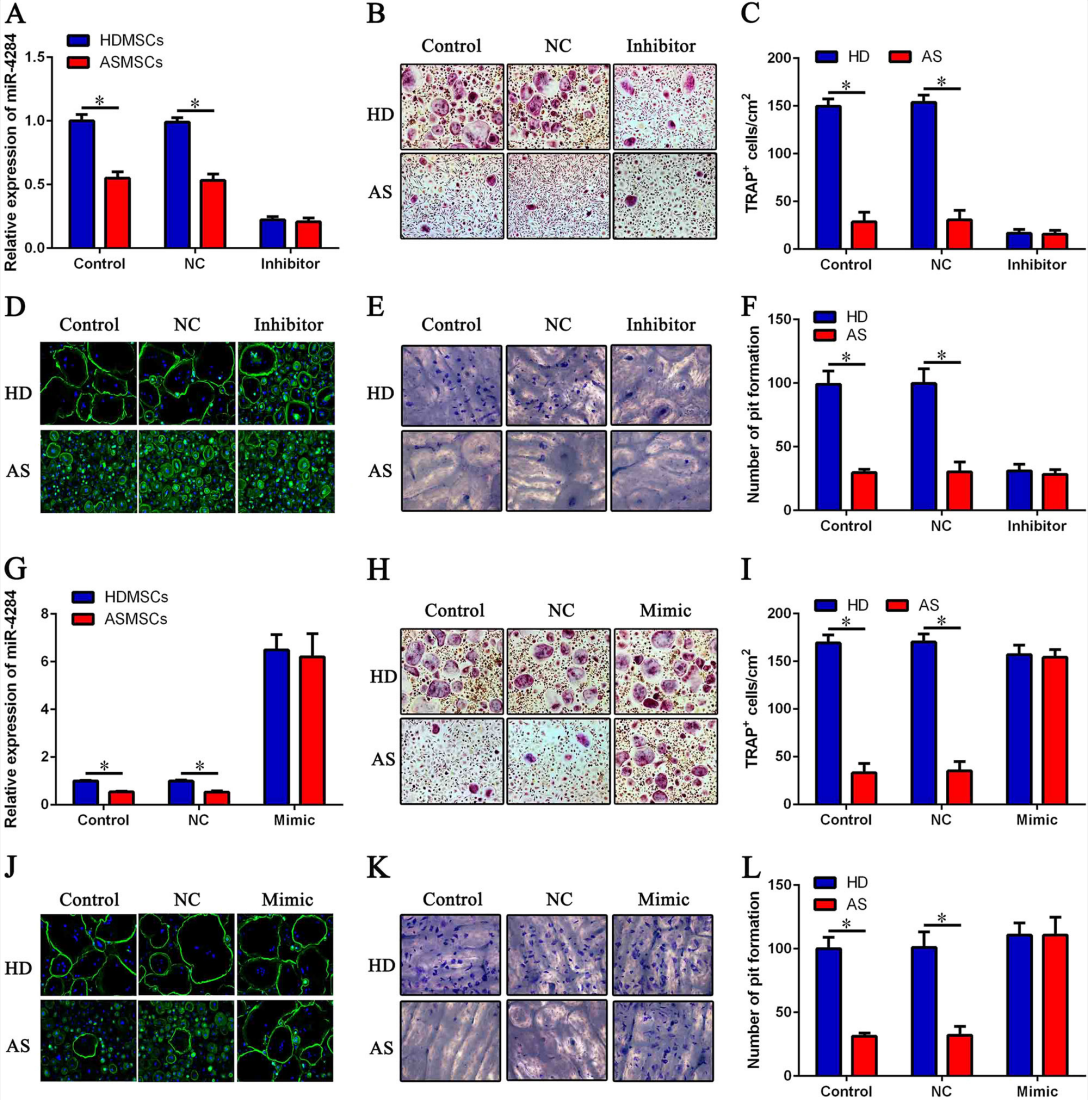
miR-4284 can regulate MSC-mediated inhibition of osteoclastogenesis. **a** Expression of miR-4284 was determined by qRT-PCR on day 3 after treatment with the miR-4284 inhibitor. **b** Representative images of TRAP staining of osteoclasts co-cultured with MSCs on day 9 after treatment with the miR-4284 inhibitor (× 100). **c** The number of TRAP^+^ osteoclasts in each well is shown. **d** Representative images of osteoclasts stained with FITC-phalloidin on day 9 (× 200). **e** Representative images of bone resorption assays on day 15 (× 200). **f** Pit formation on each slide was assessed. **g** Expression of miR-4284 was determined by qRT-PCR on day 3 after treatment with the miR-4284 mimic. **h** Representative images of TRAP staining of osteoclasts co-cultured with MSCs after treatment with the miR-4284 mimic on day 9 (× 100). **i** The number of TRAP^+^ osteoclasts in each well is shown. **j** Representative images of osteoclasts stained with FITC-phalloidin on day 9 (× 200). **k** Representative images of bone resorption assays on day 15 (× 200). **l** Pit formation on each slide was assessed. Data are presented as the mean ± SD (*n* = 30). The results represent three independent experiments. *, *p* *<* 0.05; HDMSCs, mesenchymal stem cells from healthy donors; ASMSCs, mesenchymal stem cells from patients with ankylosing spondylitis

To explore the effect of miR-4284 on osteoclastogenesis, we transfected MSCs with the miR-4284 inhibitor or mimic and found expression of miR-4284 to be reduced by at least 80% (Fig. 6a). The number of osteoclasts cultured with HDMSCs was also decreased, but there was no difference compared with that of cells cultured with ASMSCs (Fig. 6b, c). In addition, no difference in bone resorption capacity after transfection with the miR-4284 inhibitor was observed (Fig. 6d–f). Furthermore, expression of miR-4284 in MSCs was significantly increased after miR-4284 mimic transfection (Fig. 6g), and the number of osteoclasts cultured with ASMSCs recovered to the level of those cultured with HDMSCs (Fig. 6h, i). F-actin and bone resorption assays confirmed the results of TRAP staining (Fig. 6j–l). These results indicate that miR-4284 can reverse the effect of CXCL5 on the inhibition of osteoclastogenesis by MSCs.

## Discussion

In this study, we found that the capacity to inhibit osteoclastogenesis was increased in ASMSCs compared with HDMSCs. Further analyses demonstrated CXCL5 to be the main cause of the abnormal inhibition of osteoclastogenesis by ASMSCs. Bioinformatics analysis predicted that miR-4284 interacts with CXCL5, and we found that miR-4284 was downregulated in ASMSCs. miR-4284 was also shown to regulate expression of CXCL5. Indeed, there were no differences in osteoclastogenesis inhibition between HDMSCs and ASMSCs after the addition of the miR-4284 inhibitor or mimic. These data indicate that ASMSCs exhibit abnormal inhibition of osteoclastogenesis through the miR-4284/CXCL5 axis and that ASMSCs have a pathogenic role in AS by acting as a mediator of osteoclastogenesis inhibition, which may cause pathological osteogenesis in AS.

Bone remodeling in vivo depends on the balance between osteoblasts, which are derived from MSCs, and osteoclasts, which are derived from monocytes^8–10^. Either dysfunction or abnormal mutual regulation of these two cells can lead to bone homeostasis disorders, which contribute to abnormal osteogenesis in rheumatic disease^28^. Pathological osteogenesis is one of the central characteristics of AS^4^. Previously, we demonstrated that ASMSCs outperformed HDMSCs in osteogenic differentiation, one of the most important mechanisms of pathological osteogenesis in AS^7^. In addition, a recent study reported that the osteoclast differentiation ability of monocytes from AS patients is equal to that of HD monocytes when cultured in vitro^29^. Nonetheless, the mutual regulation of these two cells in AS has remained unclear. In this study, we found that the capacity of ASMSCs to inhibit osteoclastogenesis was stronger than that of HDMSCs. During osteogenic differentiation of MSCs, the capacity of MSCs to inhibit osteoclastogenesis was not significantly changed (Supplement Figure S3). From another point of view, these results highlight the importance of ASMSCs in the pathogenesis of AS, not only through their higher osteogenic differentiation capability but also through their stronger inhibition of osteoclastogenesis.

Many studies have shown that MSCs can regulate the differentiation of osteoclasts, yet their effect on osteoclastogenesis is controversial. In our study, both HDMSCs and ASMSCs were able to inhibit osteoclastogenesis. Early studies suggest that MSCs promote osteoclastogenesis^30,31^, whereas recent studies demonstrate that MSCs inhibit osteoclastogenesis^11,12^. We identified several differences among these studies. First, the types of cells cultured with MSCs differed among studies. For example, most studies showing that MSCs inhibit osteoclastogenesis, consistent with our results, utilized PBMCs^11,12^, whereas studies with opposing results used CD34^+^ hematopoietic stem cells or RAW264.7 cells in their experiments^30,31^. Second, treatment of MSCs with various stimuli has different effects on osteoclastogenesis. For example, pressure-loaded MSCs promote osteoclastogenesis, but MSCs under no pressure have the opposite effect^31^. Finally, culture conditions were also different. Some studies reporting results consistent with ours added RANKL to the medium^11,12^, though others did not^30,31^. Overall, the above factors may account for the different effects of MSCs on osteoclastogenesis. Because all the cells used in our study were isolated from humans, our results agrees with human physiological and pathological processes, and this approach may be more suitable for the research of human diseases than other types of studies. Regardless, the effect of MSCs on osteoclasts needs to be further confirmed in vivo.

To investigate the mechanism underlying the stronger inhibition of osteoclastogenesis by ASMSCs, we performed cytokine array assays to identify possible cytokines related to this process and found CXCL5 to be significantly elevated in ASMSCs, causing abnormal inhibition of osteoclastogenesis. However, mechanism responsible for MSC-mediated inhibition of osteoclastogenesis has not been fully elucidated. Oshita K^11^ found that MSCs inhibit osteoclastogenesis through osteoprotegerin production, and Audrey Varin reported that the inhibitory effect of MSCs on osteoclast formation depends on CD200 expression^12^. In our study, oversecretion of CXCL5 was the main reason for the abnormal inhibition of osteoclastogenesis by ASMSCs. These data suggest that MSCs inhibit osteoclastogenesis through a variety of cytokines instead of a single mediator. Our results, together with previous findings, may help elucidate the mechanism by which MSCs inhibit osteoclastogenesis. Previous studies have shown that CXCL5 is involved in a variety of inflammatory diseases, such as RA and pediatric ulcerative colitis^20,21^, though the effect of CXCL5 on AS is unknown. In particular, our research is the first to show that CXCL5 is elevated in AS and inhibits osteoclastogenesis. Our findings reveal a new function for CXCL5, and more roles of CXCL5 in AS should be explored in the future.

miRNAs expressed in MSCs have been shown to regulate differentiation as well as proliferation, senescence, migration, and survival^32–35^. Recent studies have also focused on the function of miRNAs in the paracrine effects of MSCs^34^. These miRNAs may modulate expression of proteins secreted from MSCs. Our study demonstrated that miR-4284 is downregulated in ASMSCs, promoting CXCL5 secretion from ASMSCs and inhibiting osteoclastogenesis. One recent study found that miR-4284 can regulate expression of CXCL5, but the researchers did not construct a CXCL5 3′-UTR mutant with a mutated seed region for the predicted miR-4284-binding sites to verify these sites^36^. Our results were consistent with their findings, and we also constructed such a CXCL5 3′-UTR mutant to confirm miR-4284 binding sites. Many studies have found that miRNAs are associated with inflammation in AS^37,38^. miR-130a is downregulated in PBMCs from AS patients, inducing inflammation by enhancing TNF-1α expression^39^. In T cells from AS patients, miRNA-199a-5p regulates autophagy and proinflammatory cytokine production by targeting RHEB^40^. However, research on the role of miRNAs in the pathologic osteogenesis of AS is limited to date. Our results indicate that miRNAs may play an important role in the pathologic osteogenesis of AS, and further studies are needed to determine the unique miRNA signatures in AS pathologic osteogenesis.

In conclusion, ASMSCs appear to have a stronger ability to inhibit osteoclastogenesis than HDMSCs via suppression of the p65 pathway, which is regulated by the miR-4284/CXCL5 axis. These results may help illuminate the mechanism of pathologic osteogenesis and provide new ideas for AS treatments. Nonetheless, our research also has some limitations, such as the lack of an animal model to study the function of ASMSCs in vivo. In addition, the reason why miR-4284 is downregulated in ASMSCs remains elusive. Further studies are needed to address these issues.

## Supplementary information

Supplementary materials

Supplementary Figure S1

Supplementary Figure S2

Supplementary Figure S3
