# Supplementary material for: Abnormal inhibition of osteoclastogenesis by mesenchymal stem cells through the miR-4284/CXCL5 axis in ankylosing spondylitis
Source: Cell Death Dis. 2019 Feb 25;10(3):188. doi: 10.1038/s41419-019-1448-x (PMC6389901; doi:10.1038/s41419-019-1448-x)
Supplement: Supplementary file 1 — Supplementary materials [file 41419_2019_1448_MOESM1_ESM.docx]

**Supplementary Table S1:** **Details of the study subjects**

|  | Healthy donors | AS patients |
| --- | --- | --- |
| Number | 30 | 30 |
| Age, years | 28.5±8.5 | 27.9±9.1 |
| Male, n (%) | 16 (53.3%) | 18 (60%) |
| HLA-B27 positive, n (%) | 0 | 30(100%)^a^ |
| Disease duration, years | NA | 6.8±2.1^a^ |
| CRP, mg/L | 2.1±0.9 | 16.3±5.2^a^ |
| ESR, mm/h | 5±1.9 | 31.8±6.3^a^ |
| BASDAI | 0.9±0.4 | 4.5±1.5^a^ |
| BASMI | 1.0±0.2 | 3.8±0.9^a^ |
| BASFI | 0.8±0.3 | 3.9±0.8^a^ |

AS, ankylosing spondylitis; HLA-B27, human leukocyte antigen B27; CRP, C-reactive protein; ESR, erythrocyte sedimentation rate; BASDAI, Bath Ankylosing Spondylitis Disease Activity Index; BASMI, Bath Ankylosing Spondylitis Metrology Index; BASFI, Bath Ankylosing Spondylitis Functional Index. ^a^, indicates *P*<0.05 compared to the healthy donor group. Data are presented as the mean ± SD.

**Supplementary Table S2: primer sequences used for qRT-PCR**

| Gene Name | Forward Primer | Reverse Primer |
| --- | --- | --- |
| GAPDH | AAGGTGAAGGTCGGAGTCAA | AATGAAGGGGTCATTGATGG |
| TRAP | TGAGGACGTATTCTCTGACCG | CACATTGGTCTGTGGGATCTTG |
| CTSK | GCAGAAGAACCGGGGTATTGA | GAAGGAGGTCAGGCTTGCAT |
| NFATc1 | TGTGCCGGAATCCTGAAACTC | GAGCATTCGATGGGGTTGGAG |
| CXCL5 | AGCTGCGTTGCGTTTGTTTAC | TGGCGAACACTTGCAGATTAC |
| ANG | CTGGGCGTTTTGTTGTTGGTC | GGTTTGGCATCATAGTGCTGG |
| GROa | CTGGCTTAGAACAAAGGGGCT | TAAAGGTAGCCCTTGTTTCCCC |
| PTX3 | AGGCTTGAGTCTTTTAGTGCC | ATGGATTCCTCTTTGTGCCATAG |
| THBS1 | AGACTCCGCATCGCAAAGG | TCACCACGTTGTTGTCAAGGG |
| U6 | CGCTTCGGCAGCACATATAC | TTCACGAATTTGCGTGTCAT |
| miR-4284 | TCGCCGACGGGCTCACATCA | CTCAACTGGTGTCGTGGAGTCGGC |
| miR-92b | TCGCCTATTGCACTCGTCCCG | CTCAACTGGTGTCGTGGAGTCGGC |
| miR-25 | TCGCCCATTGCACTTGTCTCG | CTCAACTGGTGTCGTGGAGTCGGC |
| miR-363 | TCGCCGAATTGCACGGTATCCA | CTCAACTGGTGTCGTGGAGTCGGC |
| miR-32 | TCGCCGACTATTGCACATTACTAA | CTCAACTGGTGTCGTGGAGTCGGC |
| miR-367 | TCGCCGACAATTGCACTTTAGCAA | CTCAACTGGTGTCGTGGAGTCGGC |
| miR-92a | TCGCCTATTGCACTTGTCCCG | CTCAACTGGTGTCGTGGAGTCGGC |
| miR-4284 RT Primer: CTCAACTGGTGTCGTGGAGTCGGCAATTCAGTTGAGATGGGGTG | | |
| miR-92b RT Primer: CTCAACTGGTGTCGTGGAGTCGGCAATTCAGTTGAGACAGGCCG | | |
| miR-25 RT Primer: CTCAACTGGTGTCGTGGAGTCGGCAATTCAGTTGAGTCAGACCG | | |
| miR-363 RT Primer: CTCAACTGGTGTCGTGGAGTCGGCAATTCAGTTGAGTACAGATG | | |
| miR-32 RT Primer: CTCAACTGGTGTCGTGGAGTCGGCAATTCAGTTGAGTGCAACTT | | |
| miR-367 RT Primer: CTCAACTGGTGTCGTGGAGTCGGCAATTCAGTTGAGTCACCATT | | |
| miR-92a RT Primer: CTCAACTGGTGTCGTGGAGTCGGCAATTCAGTTGAGGGAGGCCG | | |

**Supplementary Figure Legends**

**Supplementary Figure S1.** **The purity of CD14^+^ monocytes.** The percentage of CD14+ monocytes in PBMCs was approximately 11% as determined by flow cytometry. After purification using CD14 MicroBeads, the percentage of CD14^+^ monocytes was more than 95%.

**Supplementary Figure S2**. **The transfection efficiency of lentivirus in MSCs.** Both HDMSCs and ASMSCs were transfected with lentivirus effectively. GFP-positive cells were observed with the Axio Observer fluorescence microscope, and there was no difference in the transduction efficiency between HDMSCs and ASMSCs.

**Supplementary Figure S3**. **Inhibition of osteoclastogenesis by ASMSCs compared with HDMSCs during osteogenic differentiation.** Both HDMSCs and ASMSCs were cultured in osteogenic differentiation medium (consisting of DMEM with 10% FBS, 100 IU/ml penicillin, 100 IU/ml streptomycin, 0.1 mM dexamethasone, 10 mM b-glycerol phosphate,and 50 mMascorbic acid) for 7, 14 and 21 days, respectively. Then CD14+ monocytes were cultured with above MSCs in the presence of M-CSF and RANKL. (A) Representative images of TRAP staining of osteoclasts at different time points of osteogenic differentiation (×100). (B) The number of TRAP^+^ osteoclasts in each well from cultures at different time points is shown. (C) Representative images of osteoclasts stained with FITC-phalloidin at different time points (×200). (D) Representative images for bone resorption assays at different time points (×200). (E) Pit formation on each slide was assessed. (F) mRNA expression levels of TRAP in osteoclasts were determined by qPCR. (G) mRNA expression levels of CTSK in osteoclasts were determined by qPCR. (H) Protein levels of TRAP and CTSK in osteoclasts were determined by western blot analyses. (I) Quantitative data of TRAP protein levels determined by western blot analyses are shown. (J) Quantitative data of CTSK protein levels determined by western blot analyses are shown. Values are the mean ± SD of 12 samples per group. The results represent three independent experiments. *, *p* < 0.05; HDMSCs, mesenchymal stem cells from healthy donors; ASMSCs, mesenchymal stem cells from patients with ankylosing spondylitis.
