# Supplementary figures and images for: Abnormal inhibition of osteoclastogenesis by mesenchymal stem cells through the miR-4284/CXCL5 axis in ankylosing spondylitis
Source: Cell Death Dis. 2019 Feb 25;10(3):188. doi: 10.1038/s41419-019-1448-x (PMC6389901; doi:10.1038/s41419-019-1448-x)

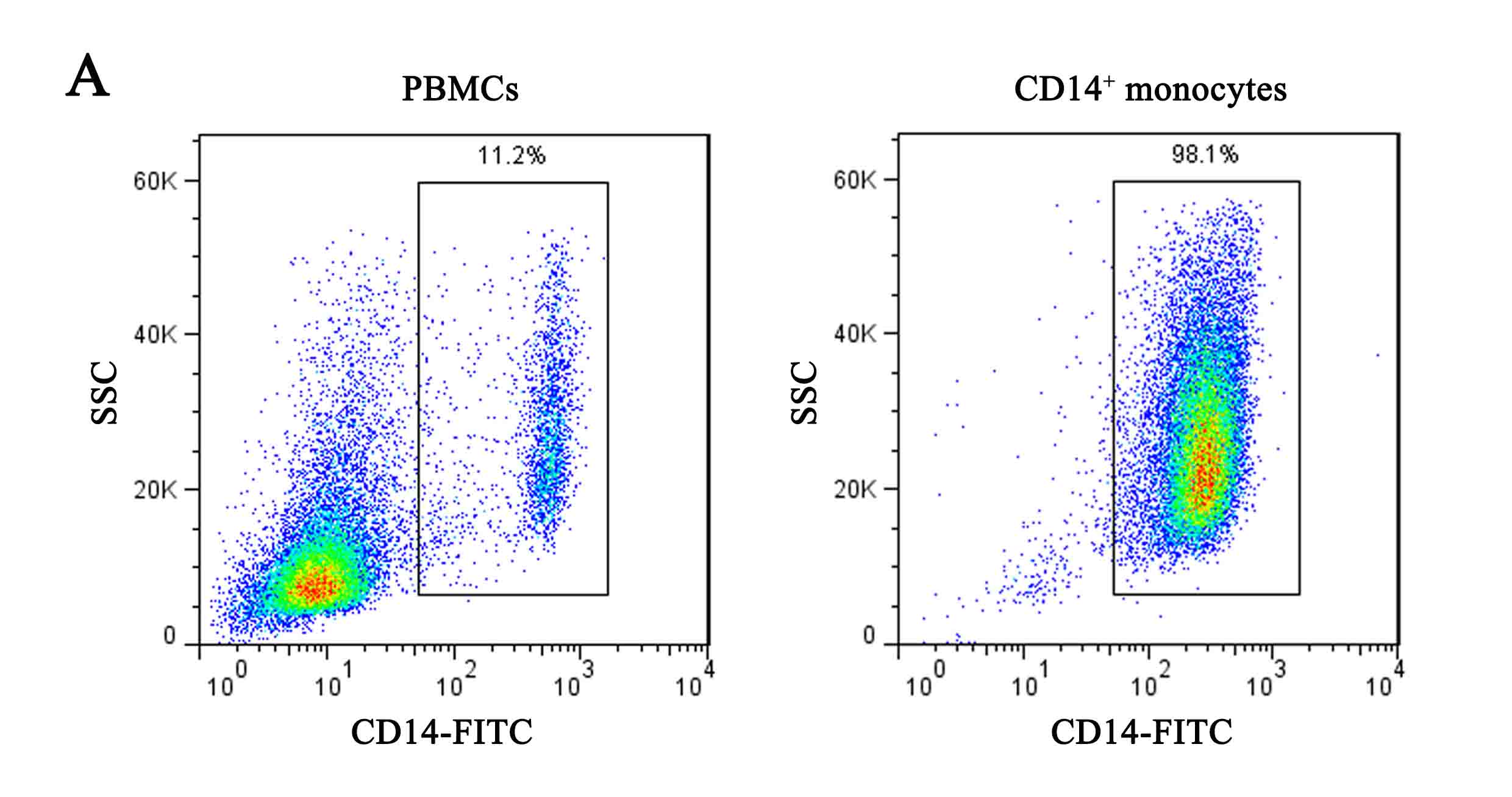

Supplement: Supplementary file 2 — Supplementary Figure S1 [file 41419_2019_1448_MOESM2_ESM.jpg]

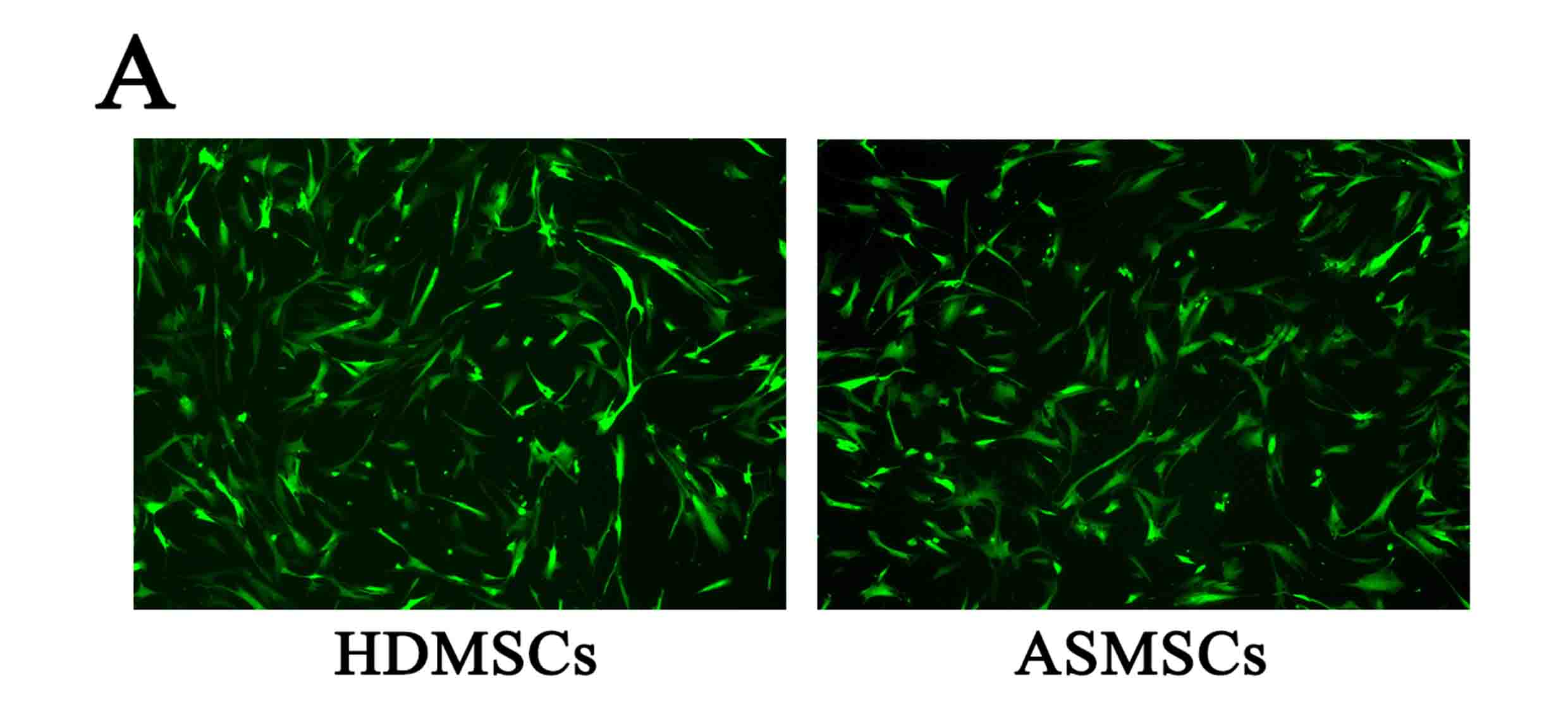

Supplement: Supplementary file 3 — Supplementary Figure S2 [file 41419_2019_1448_MOESM3_ESM.jpg]

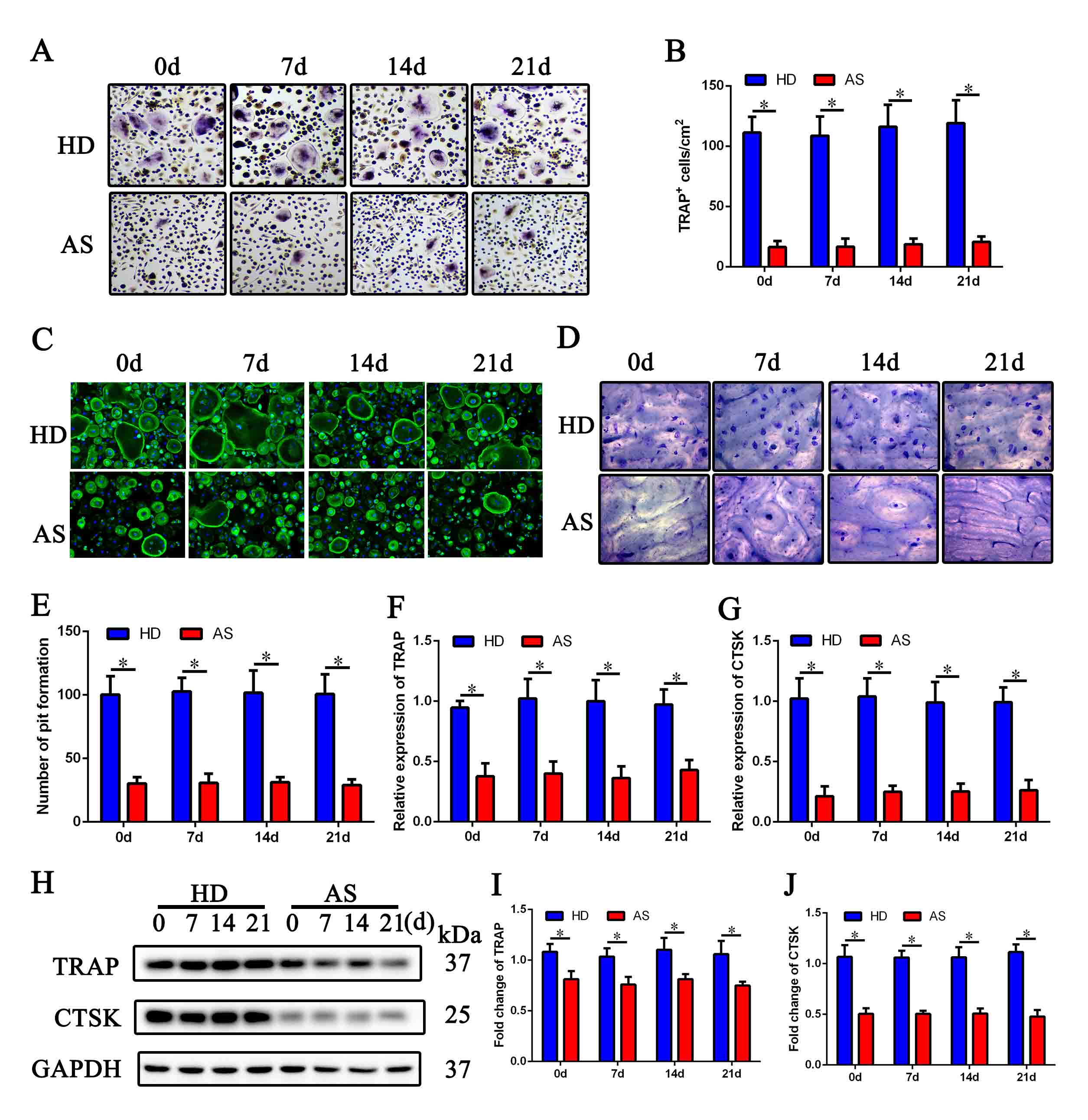

Supplement: Supplementary file 4 — Supplementary Figure S3 [file 41419_2019_1448_MOESM4_ESM.jpg]
